# Mimicking and surpassing the xenograft model with cancer-on-chip technology

**DOI:** 10.1016/j.ebiom.2021.103303

**Published:** 2021-03-25

**Authors:** Job Komen, Sanne M. van Neerven, Albert van den Berg, Louis Vermeulen, Andries D. van der Meer

**Affiliations:** a BIOS Lab on a Chip group, MESA+ Institute for Nanotechnology, University of Twente, P. O. Box 217, 7500 AE Enschede, the Netherlands; b Applied Stem Cell Technologies, TechMed Centre, University of Twente, P. O. Box 217, 7500 AE Enschede, the Netherlands; c Laboratory for Experimental Oncology and Radiobiology, Center for Experimental and Molecular Medicine, Cancer Center Amsterdam and Amsterdam Gastroenterology and Metabolism, Amsterdam University Medical Centers, 1105 AZ, Amsterdam, the Netherlands

**Keywords:** organ-on-chip, Xenograft, Cancer, Microfluidics

## Abstract

Organs-on-chips are *in vitro* models in which human tissues are cultured in microfluidic compartments with a controlled, dynamic micro-environment. Specific organs-on-chips are being developed to mimic human tumors, but the validation of such ‘cancer-on-chip’ models for use in drug development is hampered by the complexity and variability of human tumors. An important step towards validation of cancer-on-chip technology could be to first mimic cancer xenograft models, which share multiple characteristics with human cancers but are significantly less complex. Here we review the relevant biological characteristics of a xenograft tumor and show that organ-on-chip technology is capable of mimicking many of these aspects. Actual comparisons between on-chip tumor growth and xenografts are promising but also demonstrate that further development and empirical validation is still needed. Validation of cancer-on-chip models to xenografts would not only represent an important milestone towards acceptance of cancer-on-chip technology, but could also improve drug discovery, personalized cancer medicine, and reduce animal testing.

## 1 Introduction

Cancer is the leading cause of death in developed countries [1]. New treatments are continuously being developed, however the success rate in clinical trials is only 5% for novel oncology drugs [2,3]. The main reason for attrition in clinical trials is lack of efficacy[4]. A technology which could potentially improve drug success rate is organ-on-chip technology. Organ-on-chip devices are microfluidic devices with living cells that model tissue-level and organ-level aspects of human physiology [5], [6], [7], [8]. Advanced environment architecture and control of flow and solutes within these devices enables studying human disease and therapy response in more supervised fashion [6], [7], [8].

**Fig. 1:**
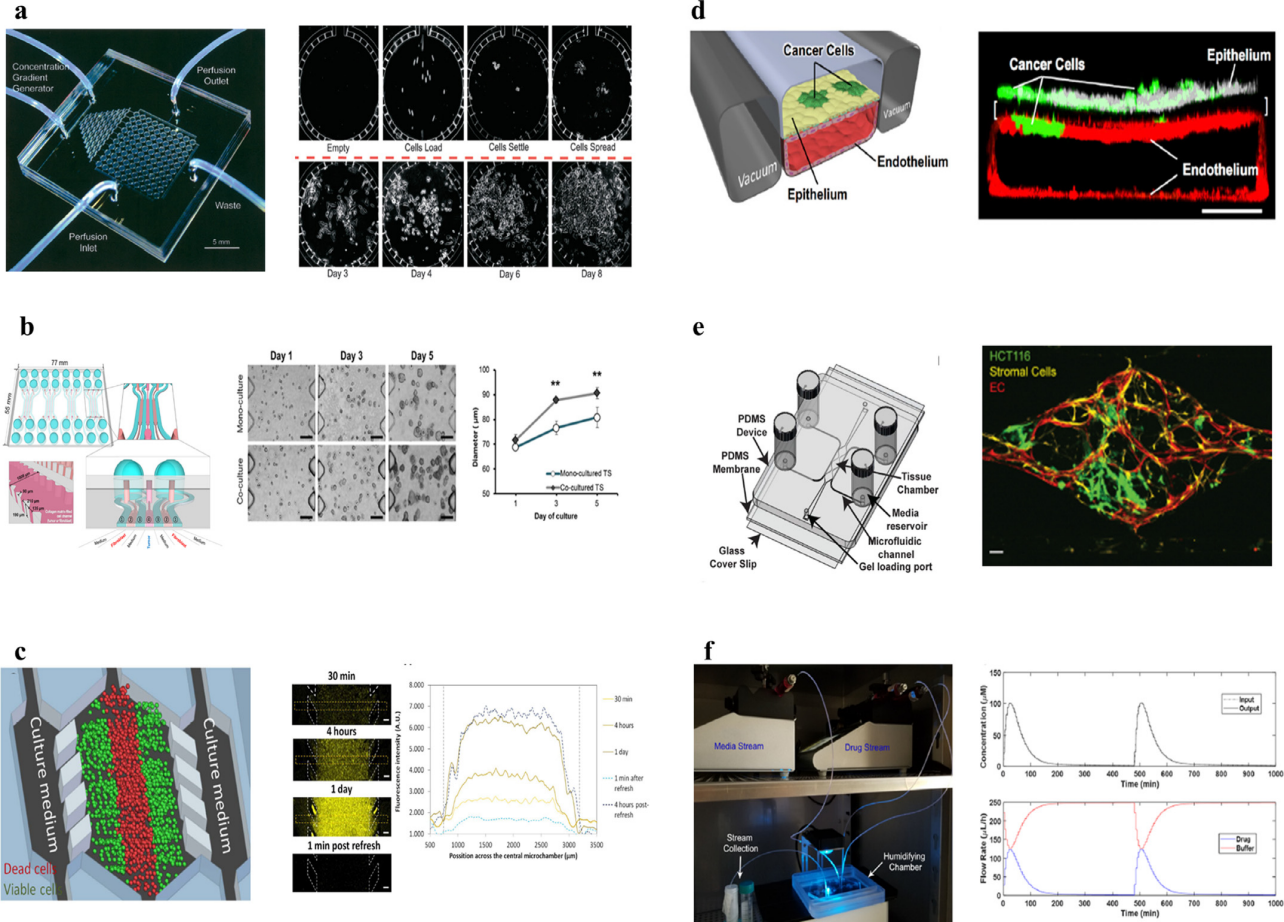
**Existing cancer-on-chip technology for mimicking cancer physiology.** (a) Parallel cancer cell culture under continuous flow on chip [13]. (b) Co-culture of fibroblasts and cancer cells in adjacent channels and the effect on cancer growth [15]. (c) A central channel for culturing cancer cells in which an oxygen and glucose gradient arises and is quantified [16]. (d) Mechanical stimulation of cancer cells via simulation of breathing on chip affects tumor invasion and growth [17]. (e) Cancer cells, fibroblasts and functional vessel formation in a microfluidic chip, which can distinguish direct drug effect and indirect drug effect via vessel formation [18]. (f) Two separate microfluidic pumps enable dynamic drug control for mimicking in vivo like drug concentration profiles in a microfluidic chip with cancer cells [19]. Reprinted with permission.

Organ-on-chip technology has also been widely used to model and study cancer, and has been reviewed elsewhere [9], [10], [11], [12]. Cancer-on-chip systems contain cancer cells and mimic one or several aspects of tumor physiology, for example cell culture, nutrient gradients, interaction with support cells, dynamic drug concentrations and drug effect, angiogenesis or mechanical stimulation of cells (Fig. 1) [13], [14], [15], [16], [17], [18], [19], [20]. Such ‘cancer-on-chip’ technology development has mainly focused on directly mimicking human cancer response, as this is ultimately the goal and would allow to bypass (imperfect) animal testing [21]. However, it is extremely challenging to make systematic comparisons between experimental cancer-on-chip models with clinical data, due to the experimental nature of the models, the heterogeneity of cancer in humans and the need for expensive clinical trials. This lack of systematic comparison is hampering the validation and subsequent acceptance of cancer-on-chip models in preclinical and regulatory drug testing.

**Fig. 2:**
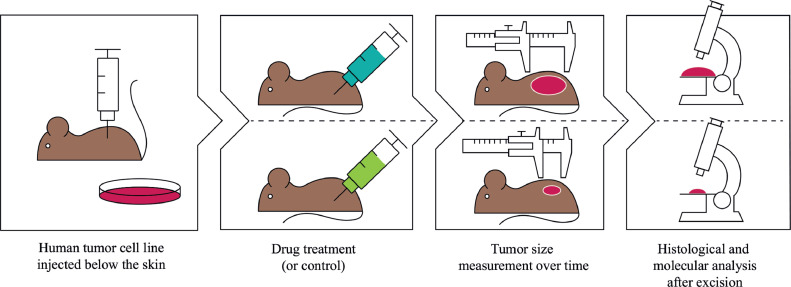
**The human cancer cell line subcutaneous xenograft model for testing drug efficacy.** Human cancer cell lines are injected below the skin of the mouse. The control group does not receive drug, while one or more groups of mice receive drug treatment(s). The primary outcome parameter is growth inhibition by the drug, for which tumor size is measured over time, often externally with calipers. After the tumor reaches a certain size the mice are euthanized. Drug effects on the tumor are further analyzed on a tissue level or molecular level (e.g. DNA, proteins).

**Fig. 3:**
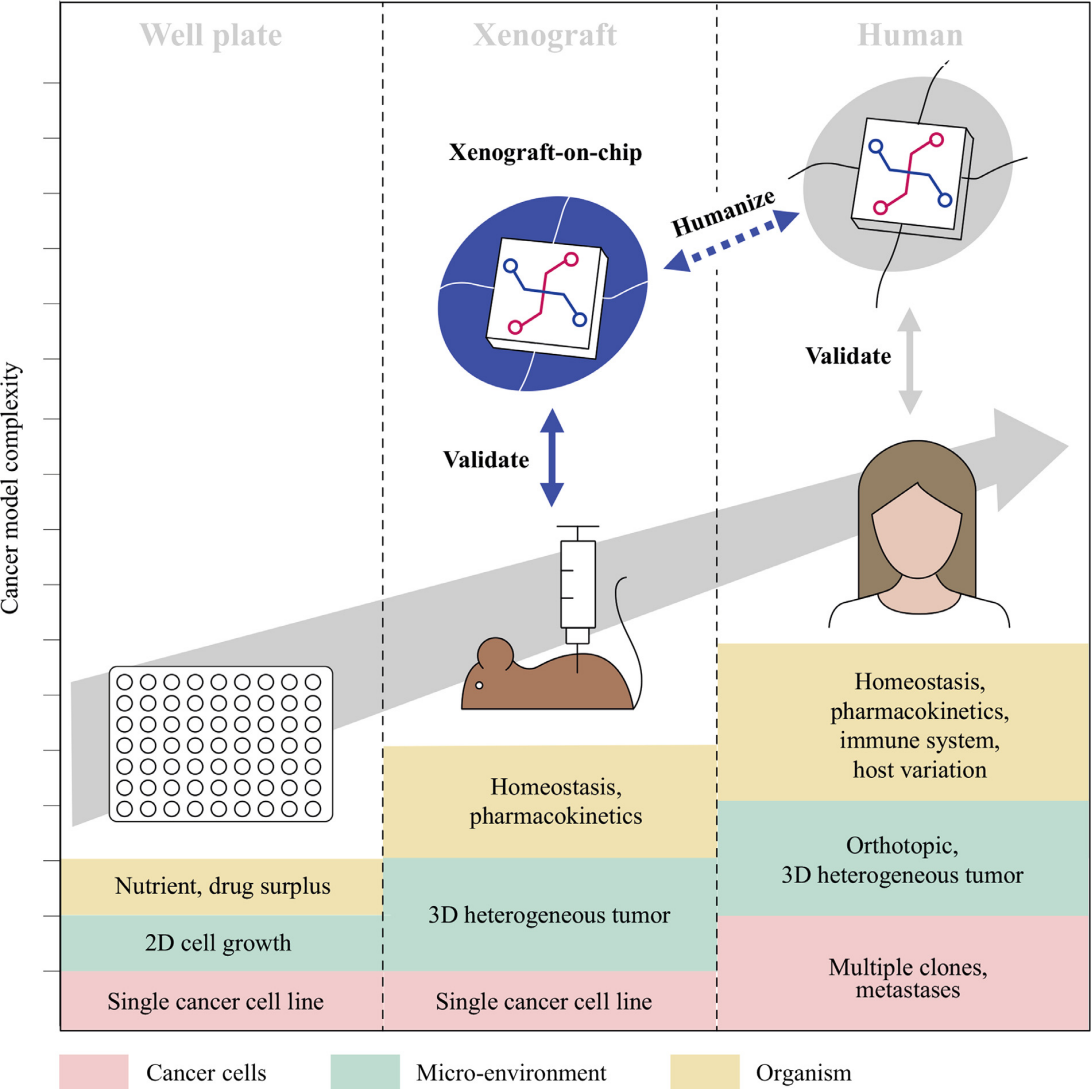
**Validation of cancer-on-chip tumor technology with animal models provides a stepping stone towards mimicking human cancer.** Once homeostasis, pharmacokinetics, and a 3D heterogeneous tumor micro environment can be reliable modeled on-chip, further human cancer complexity can be added such as multiple clones, metastases, orthotopic location, an immune system, and host variation.

Therefore we propose to first validate cancer-on-chip technology on animal models such as the mouse xenograft model (Fig. 2). The xenograft model is the gold standard for testing the efficacy of a novel oncology drug, before progressing into (human) clinical trials. In this model human cancer cells are implanted in a mouse and the effect of drugs on tumor growth analyzed. The xenograft model shares several important characteristics with human tumors; the model entails a vascularized, three dimensional tumor, growing in a living organism with homeostasis, drug tolerance and metabolism. As the model lacks certain characteristics of human tumors such as intra- and inter tumor heterogeneity, human stroma, human pharmacokinetics, and an immune system [22], the xenograft model provides an intermediate step towards the complexity of human cancer (Fig. 3). Due to the importance of xenograft models for progressing to human trials, every year hundreds of thousands of animals are used in experiments where the implanted cancer cell lines are known, animals are virtually identical (inbred), and drug responses are registered. This wealth of data from well-characterized *in vivo* models provides an opportunity for the validation of cancer-on-chip models.

Once cancer-on-chip systems reliably mimic the drug response in animal cancer models, these systems can surpass the xenograft model in several ways. The lack of certain human characteristics reduces the predictive power of basic xenograft models for efficacy of novel drugs in humans [2,3]. With further development, cancer-on-chip systems can mimic and be compared with more advanced xenograft models, such as those with cancer cell implantation in the organ of origin (‘orthotopic’), those with addition of immune system components[23], and patient-derived xenografts (PDX) [24,25]. Also, with their miniaturized nature, more cell lines can be tested in cancer-on-chip models, thereby providing a sample more representative of intra- and inter-tumor heterogeneity in humans. Furthermore, the well-controlled nature of cancer-on-chip models could reduce the variation that is typically observed in experiments with mice, due to e.g .inter-individual differences and technical variation in drug administration and tumor size measurements. With these steps the ‘pre-clinical’ to clinical gap in drug discovery can be further reduced. Cancer-on-chip models could also be employed for personalized medicine as an alternative to successful but difficult and expensive individual patient-derived xenografts in mice [26], [27], [28].

Hence mimicking cancer xenograft models represents a well-defined, first step for validation of cancer-on-chip systems to mimic human cancer response in a step-wise manner. Furthermore the validated cancer-on-chip systems could provide an improved drug discovery tool and reduce animal testing. In this review we evaluate the readiness of organ-on-chip technology for simulating the xenograft model drug response by first describing the murine xenograft model biology, and subsequently the available organ-on-chip technology for mimicking these characteristics.

## 2 Cancer xenograft biology

In the xenograft model, human cancer cells are implanted in an immunodeficient mouse. Subsequently a drug or drug combination is administered. The primary outcome parameter of a xenograft model for testing drug efficacy is the growth of the treated tumor versus control (Fig. 2).

Typically, xenograft tumors grow from 100 mm^3^ (6 mm diameter) to 1000 mm^3^ (12 mm diameter) in several weeks. The growth rate often declines with larger tumor sizes, as opposed to exponential growth in (2D) *in vitro* experiments [29], [30], [31], [32]. Tumor growth inhibition varies per drug and implanted cell line.[33] Often there is a decrease in growth under treatment, however tumor volume decrease also occurs [33,34]. The effect of therapeutics can be directly on tumor cells, or indirectly via the microenvironment such as the inhibition of vessel formation, or both [34], [35], [36].

Biological factors implicated in tumor growth are potential candidates for incorporation into chip-systems. A selection of these factors is discussed below, and an overview of example values for these factors is provided in supplementary table 1.

### 2.1 Human cancer cell lines used in xenografts

Cell lines that have been often used in *in vivo* studies provide the most data for validating the chip system. Examples of often used cancer cell lines for xenografts are HCT116 (colorectal), MCF7 (breast) and A549 (lung), or more broadly, the NCI60 panel, which consists of 60 well-characterized tumor cell lines. These cell lines have been grown *in vitro* for numerous passages, leading to fast doubling times and loss of heterogeneity and original tumor characteristics [37]. Nevertheless the level of cell differentiation and retention of original tissue structure after implantation differs per cell line [38]. Alternatively cells can be used that have been harvested from a patient, and grown for multiple generations in immunodeficient mice (‘Patient Derived Xenografts’, PDX). These cells retain more of their histologic and molecular characteristics, and intra-tumoral heterogeneity than *in vitro* passaged cell lines[24].

### 2.2 Xenograft tumor microenvironment components and gradients

The human cell lines will be injected in the mouse. Subcutaneous (below the skin) implantation is the most commonly used location for tumor xenografts due to its high engraftment success rate, rapid tumor growth and ease of external caliper measurement. Subcutaneous xenografts tend not to spread to other parts of the body [39].

Besides cancer cells a subcutaneous xenograft consists of extracellular matrix and stroma cells [40,41]. The extracellular matrix (ECM) consists of proteins (collagen, fibronectin) and proteoglycans (e.g. hyaluronic acid), of which collagen is the most abundant [42,43]. These molecules form a fiber-like network by crosslinking. The extracellular matrix can affect drug diffusion, cellular behavior and response to drugs [44]. The exact composition of the extracellular matrix varies per cell line, and the specific ‘matrisome’ has been described for several xenografts [43,45]. The ECM is dynamic, e.g. matrix metalloproteinases can break down ECM[42] and ECM components are produced by both the tumor cells and the stroma cells [43]. The amount of ECM differs between human tumor types, ranging from 20–90% [45,46]. Although xenografts generally have less stroma than primary human tumors[40,47], the amount of stroma in xenografts also depends on the cell line [45,48].

The most prevalent stroma cells in xenograft tumors are (murine) fibroblasts and endothelial cells. As with the ECM, the amount of fibroblasts and endothelial cells combined can differ per cell line in the range of 10–80%, with the difference driven by fibroblasts [40,49]. Besides producing extracellular matrix, (cancer associated) fibroblasts (CAFs) are implicated in tumor growth, invasion, angiogenesis and chemoresistance via cell-cell contact and paracrine signalling [29,[50], [51], [52]]. Fibroblasts and extracellular matrix can also affect tumor growth via increasing the pressure in a tumor, either directly or via collapsing vessels [53,54].

When tumors grow beyond 1 mm^3^, small blood vessels (capillaries), formed by endothelial cells, are needed for oxygen and nutrient supply [55,56]. Tumor vascularization is less structured than vascularization in normal tissue, e.g. shunts and abnormally wide vessels exist, which contributes to poor tissue perfusion and can lead to central or diffuse necrosis [57]. Hence lagging functional vascularization is a possible reason of the declining growth rate of tumors [56]. As nutrients, oxygen and therapeutics are supplied via the capillary, a concentration gradient arises away from the capillary. The concentration gradient depends on the concentration supplied, mass transfer (diffusion, convection), and binding and consumption in the tissue. Due to the higher interstitial pressure in the tumor, convection from the vessel into the tissue is lower, thereby limiting mass transfer mostly to diffusion.[9] Besides a gradient away from the capillary, the periphery of the tumor typically is better perfused [57]. Resulting gradients for oxygen and glucose range from the arterial level to ~0 [58], [59], [60], [61], [62]. As the capillary supply of nutrients and oxygen is crucial to tumor growth, a central capillary with a cylinder of tumor tissue (‘tumor cord’) can be seen as a functional unit of a tumor, alike a liver globule (acinus) or kidney nephron.[57] Indicative dimensions of the tumor cord are a capillary length of 0.2–0.5 mm [63],[57] with a tumor cylinder radius of ~50–100 µm.

To summarize the tumor micro-environment, the typical xenograft tumor contains stroma cells and extracellular matrix besides cancer cells, and nutrient and oxygen concentrations decrease in distance from tumor vessels.

### 2.3 Host organism: mice homeostasis and pharmacokinetics

Mice maintain steady internal conditions (‘homeostasis’) of temperature, oxygen and nutrients. which in several aspects are comparable to humans and different from *in vitro* conditions. Although body temperature is approximately 37 ˚C[64], which is equal to an incubator, mice have blood glucose of 6–7 mM[65], which is 1.5-3× lower glucose than most cell culture media, while being more diverse in amino acids [61].

Mice have arterial blood levels of 90–100 mmHg oxygen pressure, with >95% of oxygen bound to hemoglobin [66,67]. Hence this is different from *in vitro* conditions with approximately 140 mmHg oxygen pressure and no hemoglobin bound oxygen [68].

Besides maintain homeostasis, mice affect the drug concentration over time. After an initial peak drug concentration in the blood due to drug administration, the concentration declines due to distribution, metabolism and excretion [69,70]. The concentration profile can be described by several parameters, such as the maximum concentration (C_max_), drug half life (t_1/2_), and the drug concentration integrated over time (Area Under the Curve, AUC). However the exact relation between these parameters and drug efficacy are usually not known [19,35]. Therefore the concentration profile over time cannot easily be substituted by a single constant dose or pharmacokinetic parameter. In blood drugs are also protein bound to a different degree, which reduces the amount of free drug which is pharmacologically active [71]. Furthermore mice can receive single, or multiple rounds of drug treatment, ranging from once per week to once per day [72,73].

### 2.4 Experimental variation in xenograft models

The aforementioned biological characteristics could influence drug response in the xenograft model and therefore might be relevant for chip-systems mimicking the drug response. However several other characteristics of the xenograft model primarily lead to variation in the outcome without biological relevance.

Variations arise for multiple reasons. Although mice typically used for xenograft studies are inbred and therefore virtually genetically identical, different strains, gender or microbiome could still lead to variation. Furthermore housing or conditions inducing stress could affect tumor growth [74], [75], [76]. Also the subcutaneous injection of tumor cells can give rise to differently sized and shaped tumors. Injection of drugs into the tail vein or intraperitoneal can fail. External measurement of an irregularly shaped tumor, placed under the skin, with calipers in two directions on a millimeter scale gives rise to measurement errors. This variation can lead to false positive and negative results. This complicates mimicking the outcome of the xenograft model, and could require taking the average response of several studies, however also provides an opportunity for cancer-on-chip systems to provide a more statistically robust outcome then xenograft models.

## 3 Capturing essential xenograft biology in cancer-on-chip models

From the previous sections it might seem complexity and variation in xenograft tumor characteristics is infinite, and would lead to impossible technical requirements for cancer-on-chip models to capture the response to drug treatment. However the 6-12 mm diameter tumor can be reduced to a collection of functional units, or ‘tumor cords’. Furthermore, an educated guess to rank the tumor characteristics most relevant to drug response, and a subsequent selection, could lead to a cancer-on-chip model that is able to predict the in vivo drug response. For example an initial model could match the cell line, incorporate gradients of oxygen, nutrients, and drug, and apply drug concentrations found in vivo. From such a relatively simple ‘starting point’ additional characteristics such as cancer associated fibroblasts, angiogenesis, and/or pressure, can later be added. These characteristics can be added for testing in general, or for testing of specific drugs and cell lines.

## 4 Cancer-on-chip tumor growth correlated with xenograft data

**Fig. 4:**
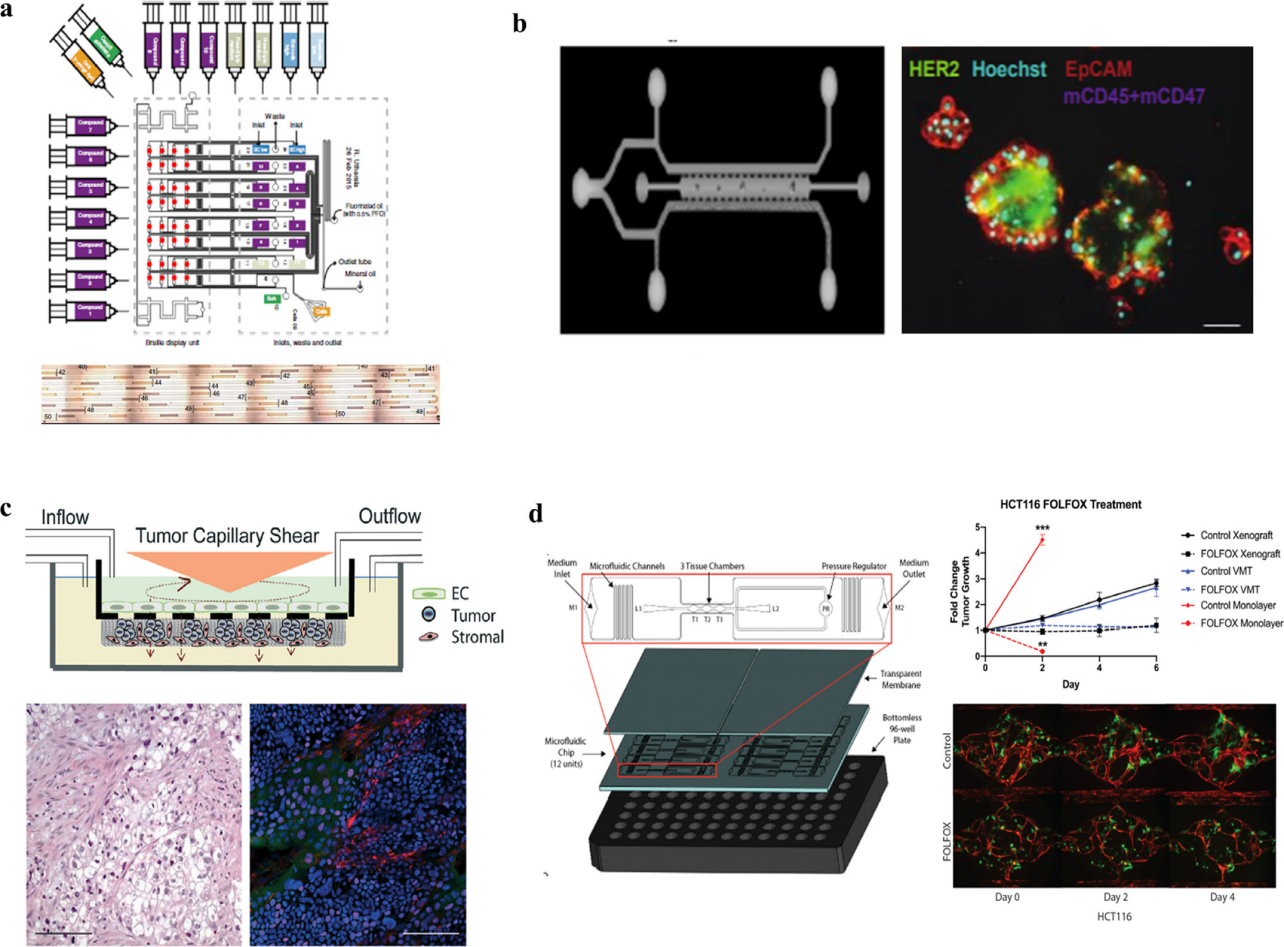
**Cancer-on-chip studies with a comparison to xenograft cancer growth.** (a) Parallel platform to test drug combinations on cell plugs with a caspase read out. Increased caspase correlated to growth inhibition in xenografts [77]. (b) A separate channel for introducing PDX spheroids and supplying medium and drugs, with a live/dead stain readout which correlates to growth inhibition in xenografts [80]. (c) Multi cell layers consisting of endothelial cells, tumor and stroma cells in a micro-physiology system. Cancer cell growth inhibition and molecular analysis on-chip is comparable to xenografts [78]. (d) Drug and nutrients are provided to cancer cell clusters (green) via self forming vessels (red). On-chip growth and growth inhibition is comparable to xenograft growth and growth inhibition for first line colorectal cancer therapy (FOLFOX) [81]. Reprinted with permission.

The validity of such a ‘reductionist’ approach in the design of cancer-on-chip models is illustrated by the first direct comparisons of tumor growth in cancer-on-chip models to xenograft cancer growth. These studies are relatively scarce, but show promising results (Fig. 4) [28,[77], [78], [79], [80], [81]].

Drug-induced cancer cell death in a multiplexed microfluidic device was correlated to growth inhibition in a xenograft for pancreatic cancer cell lines and primary cells [77]. A microfluidic platform was created for applying 56 simultaneous drug combinations times 20 replicates to fluid plugs which contained ~100 tumor cells [77]. Drugs were added at a constant, uniform concentration and incubated for 16 h. Activation of caspase 3, an apoptosis protein, was used to quantify drug efficacy on two pancreatic cancer cell lines. Drug efficacy found in the microfluidic device was subsequently compared to growth inhibition in subcutaneous xenografts. Effective drug combinations found in the microfluidic device also led to growth inhibition in xenografts [77].

Cancer-on-chips with less parallel processing but a higher degree of micro-environment complexity were used in different studies. Spheroids have multiple cell layers and thereby induce a gradient of nutrients and drugs from the outside in. When spheroids derived from PDX bladder cancer cells were cultured in microfluidic chambers, they could be exposed to fixed concentrations of drugs for multiple days [28]. Interestingly, in both the cancer-on-chip microchambers and in mice, combination therapy of cisplatin and gemcitabine inhibited growth, whereas individual agents did not. Similarly, when PDX lung tumor spheroids were cultured in a microfluidic device and exposed to epithelial growth factor receptor (EGFR) inhibitors, the decrease in viability correlated well with xenograft growth inhibition of different drug (combinations) [80].

Increasing complexity can be included in the cancer-on-chip models to capture even more aspects of tumor biology. For example, a microfluidic chip with multiple layers of patient derived pancreatic cancer cells with fibroblasts on one side of a membrane and endothelial cells on the other side has been reported [78]. Morphology of the microfluidic cell cultures showed disordered growth of tumor and stroma cells, comparable to xenografts. Upon perfusion with average drug concentrations found in mice, the transcriptome profile obtained with RNAseq suggested a stronger correlation with xenograft gene transcription, compared to 2D *in vitro* gene transcription [78]. Furthermore, growth inhibition for several drugs was similar to growth inhibition in xenografts of the same cell lines [78,82].

Further sophistication of the tumor micro-environment was achieved in a microfluidic chip with self forming vessels. The vessels provide nutrients and drugs to cancer cell clusters embedded in extracellular matrix containing fibroblasts [81]. Two standard colorectal cancer cell lines which cover different archetypes of colorectal cancer were used. Vessels perfusion was achieved with a pressure gradient over the microfluidic chamber in which both the vessels and tumor cells are located. Computational modeling showed comparable vessel flow speed as in capillaries. As test drugs, 5-fluorouracil, leucovorin, and oxaliplatin, which are first line treatment for metastatic colorectal carcinoma, were chosen. Administered drug concentrations were based on maximum concentrations and drug half life found in men. A single cycle was applied and growth evaluated after 6 days. Tumor growth of both the control and treatment group in the chip was similar to xenograft growth, whereas two-dimensional growth differed significantly. Furthermore RNA analysis indicated a higher similarity between xenograft and on-chip tumors than with two-dimensional cell culture.

These studies clearly illustrate the potential of cancer-on-chip technology to mimic the xenograft drug response. Especially the more complex systems (almost) cover the tumor characteristics chosen as a ‘starting point’: cell lines are matched, the microenvironment consists of multiple cell layers that generate solute gradients, there is a constant supply of nutrients and oxygen, and drug concentrations are based on *in vivo* concentrations. Furthermore a drug response which correlates to the *in vivo* drug response was observed.

## 5 Improving correlation to xenograft biology with cancer-on-chip development

Further mimicry of the xenograft tumor than already achieved by the examples listed above could be realized by incorporating dynamic drug control, controlled solute gradients and physiologic homeostasis. Beyond that, more advanced xenograft characteristics such as fully functional vessel growth can be incorporated. In the broader cancer-on-chip literature solutions exist for these parameters, although none have implemented all of them as of yet.

Nutrient and oxygen homeostasis can be achieved by applying continuous flow to cell culture[13]. Cell medium used usually contains supraphysiological levels of glucose and other nutrients, however medium can be modified to be more *in vivo*-like for known medium constituents[83]. Furthermore ambient oxygen tension is typically higher than *in vivo*, however because of the absence of hemoglobin the amount of oxygen per medium volume is approximately forty times lower. For oxygen control, oxygen scavengers, adjacent gas supply channels or gas impermeable chips limiting the gas supply have been used [84]. To increase the oxygen concentration in medium artificial oxygen carriers such as perfluorocarbons can be used [85]. Both oxygen and glucose demand and supply can be modeled based on *in vitro* cellular consumption and numerical simulations[86,87].

Dynamic, *in vivo*-like, drug concentrations have been created in microfluidic chips based on existing pharmacokinetic data. Drug concentrations can be controlled by multiple pumps, or channel geometry [[19], [20], [21],^88^]. An even more ambitious approach is to use body-on-chip technology to generate the pharmacokinetic profile of the drug [89,90]. Absorption of drugs by often used chip materials such as silicone rubbers is a known issue, and can be circumvented by using different materials, or by adjusting the dosage and continuous flow [21,91].

Micro-environment nutrient and drug gradients arise from the outside of the tumor to the inside due to diminished blood flow, and radially outward from the tumor capillaries. Ideally the gradient on-chip matches the absolute values, and steepness of *in vivo* values for relevant solutes such as oxygen, glucose, other nutrients and drugs. To create gradients on chip multiple approaches are feasible. One approach is to create different concentrations in separate channels by using a gradient generator and parallel channels. Double layers of gradient generators allow for independent oxygen and chemical gradient generation in a single chamber [92]. A gradient can also be created across a single channel with cells consuming nutrients [16]. Multiple stacked cell layers, for example in spheroids, also generate gradients across the cell layers, which can be quantitatively evaluated [86].

Different degrees of vessel incorporation and functionality into the model are possible based on existing microfluidic technologies. An endothelial cell layer could increase the validity of the model by providing a barrier to drug diffusion [78]. Furthermore, self-forming vessels can be created on-chip to both provide cancer cells with nutrients and drug and indicate the effect of drugs on vessel formation [18]. Although the self-forming vessels are the sole source of oxygen and nutrients, the differently sized cancer cell (cluster)s are still also dependent on nutrient diffusion from the empty extracellular space, instead of a central capillary in a tumor cord. Vessels which grow within a compact tumor, are the sole provider of nutrients to the compact tumor, and allow the tumor to grow larger than an avascular tumor, have not been achieved yet, neither on chip nor in spheroids.

Despite the availability of technology to mimic separate aspects of xenograft tumor physiology on-chip, integration is not trivial. In general adding complexity reduces scalability, e.g. the use of microfluidic pumps for dynamic drug concentration control requires a separate technical solution to scale up [93,94]. Furthermore the preferred solution for one aspect might complicate control of another. For example oxygen control is often established on chip by using oxygen permeable materials. However potential absorption and desorption of drugs in the oxygen permeable material could require additional attention to the drug administration schedule. Also reliance on biology for recapitulating certain unknown parameters, such as multiple cell layers for drug transport through and around cells, could increase variability and distort other parameters such as oxygen availability, requiring compensation.

Hence which biological parameters are crucial and whether the engineering solution suffices requires careful consideration. The aforementioned, relatively simple, ‘starting point’ tumor characteristics, together with the technologies mentioned in the previous two paragraphs, could aide in designing such as a system.

## 6 Future validation of cancer-on-chip systems using *in vivo* xenograft data

There is considerable interest in using organ- or cancer-on-chip for drug discovery, as illustrated by academic, industry and regulatory publications[19,21,95,96]. However to obtain an established role in drug discovery cancer-on-chip systems need empirical validation. For initial validation of cancer-on-chip models commonly used cell lines are suitable. Although primary and PDX cells have a higher translational value, these cell lines are less stable and homogeneous and less data on growth inhibition is available. A starting point could be cell lines from the NCI-60 panel [97]. Initial drugs tested are preferably drugs approved for use in the clinic, as there is likely efficacy and hence an effect to measure, and the most data available as these drugs have gone through the approval process. Furthermore, efficacy should preferably not depend on active metabolites, and drugs to have a direct effect on cancer cells instead of indirect effects via e.g. angiogenesis inhibition. Hence the drug-cell line combination for initial validation should allow for the relatively simple ‘starting point’ tumor characteristics. The approach of validating a chip system with a standard cell line and approved drugs to a xenograft has been used [81]. Further examples of common combinations of cancer-cell lines and drug are provided in the Supplementary Material, as well as a guide to obtain growth inhibition data and accompanying pharmacokinetic data. As the search strategy and access to articles might lead to a bias, it is advisable to discuss the findings with researchers who conduct relevant xenograft studies. Also there is considerable statistical variation in mice xenograft data, it is advisable to start with drug dosages with a significant effect and use an average of growth inhibition found across studies.

Crucial is validation on the primary outcome parameters of control growth and growth inhibition by the drug applied. For further validation of the technology, histopathology and molecular analyses on gene expression and/or protein levels of cancer-on-chip models could be compared to the *in vivo* tumor. Moreover face validity as judged by xenograft experts could aid in determining the validity. A minimum validation of several cancer cell lines with several drugs and dosages will be required. Once there is initial empirical validation, a multi-site, blinded trial comparing growth inhibition of multiple compounds in cancer-on-chip models to *in vivo* growth inhibition could be conducted. A recently conducted trial on the predictive power for cardiotoxicity of three dimensional engineered heart tissue could serve as an example. In this trial a set of 36 compounds was used, of which 8 were training compounds, and 28 were blinded test compounds[98].

After the validation on established cell lines and drugs, the model could be employed in the drug discovery process complementary to the *in vivo* model, e.g. to test the efficacy of novel compounds on different cell lines and with different administration schedules. If the cancer-on-chip model suggests a superior drug (schedule) exists, this can be retested in mice, to both act as a further validation of the model and to improve lead (compound) selection and optimization in drug discovery. With increasing validation the cancer-on-chip model can progressively substitute the use of mice. Nevertheless, though significantly reduced, animal models will remain needed for both toxicity and pharmacokinetics analysis, until ‘body on-chip’ devices have been tested sufficiently extensive to correctly predict (tolerable) drug concentrations and metabolism [89,90].

Once the chip-system has been validated for xenograft drug responses the system can be made more human-like. For example by adding factors such as primary or PDX cells, human stroma, pharmacokinetics, and immune components. Also the chip-system can be used as substitute for PDXs in mice for individual patients to personalize treatment [27].

## 7 Conclusion and outlook

Mimicking human cancer on a chip would allow for personalized medicine and improve drug discovery, however has proven elusive. Mimicking a murine subcutaneous xenograft in a cancer-on-chip system would be an important and verifiable intermediate step towards human cancer-on-chip. Moreover chip systems could then surpass the mice xenograft in scale and robustness, immediately improving drug discovery and reducing animal testing.

A human cancer xenograft in a mouse is arguably the most simple *in vivo* tumor, however it is still a vascularized three-dimensional tumor with stroma growing in a living animal. Despite the theoretically infinite complexity and variation, an educated guess on the most relevant tumor characteristics for predicting therapy response can be made as a starting point, and complexity added where needed.

Current organ-on-chip technology is able to model many of the relevant biological parameters of the cancer cells, microenvironment, and host that are typical of xenografts. Some advanced organ-on-chip systems have incorporated several aspects and shown promising correlations to xenograft response. However further technical integration and empirical validation to *in vivo* tumor growth with common cell lines and drugs is needed.

Overall, we conclude that the development of a cancer-on-chip system that is able to mimic the xenograft drug response should be feasible in the short term, and the benefits would be vast. Therefore validation of cancer-on-chip models to xenograft models warrants a significant effort from the cancer-on-chip scientists, either as separate research or incorporated into regular (human) cancer-on-chip development.

## 8 Search strategy and selection criteria

Data for this review were identified by searches of PubMed, and references from relevant articles using the search terms “xenograft”, “microfluidic”, “cancer”. For the biology section searches were conducted on “xenograft” and the different paragraph topics, and for the microfluidics section paragraph topics were combined with “microfluidic”.

## Contributors

J.K. – Conceptualization, Investigation, Methodology, Visualization, Writing – original draft; S.M.N. - Investigation, Writing – original draft; A.B. – Funding acquisition, Supervision, Writing – review & editing; L.V. – Funding acquisition, Supervision, Writing – review & editing; A.D.M. – Conceptualization, Methodology, Supervision, Visualization, Writing – review & editing.

## Funding

This work was supported by the Netherlands Organ-on-Chip Initiative (NOCI), an NWO Gravitation Project funded by the Ministry of Education, Culture and Science of the Government of the Netherlands, under Grant 024.003.001.

## Ethics committee approval

Not applicable.

## Declaration of Competing Interest

The authors declare no conflict of interest.

## Appendix Supplementary materials

Image, application 1
