# Supplementary material for: Mimicking and surpassing the xenograft model with cancer-on-chip technology
Source: eBioMedicine. 2021 Mar 25;66:103303. doi: 10.1016/j.ebiom.2021.103303 (PMC8024912; doi:10.1016/j.ebiom.2021.103303)
Supplement: Supplementary file 1 [file mmc1.docx]

**Supplementary material**

**Supplementary table 1.** Example values for xenograft tumor parameters, which can serve as a input for cancer-on-chip requirements.

| **Topic** | **Parameter** | **Example values cancer xenografts** |
| --- | --- | --- |
| Tumor growth | Measurable macroscopic diameter | 6-12 mm^99^ |
|  | Size increase | Exponential – linear/ Gompertzian, cell line dependent^31, 32^ |
|  | Experiment duration | Several weeks^73^ |
|  | Drug growth inhibition | 0-100%; drug dependent^73^ |
| Cancer cells | 2d growth speed | 35 (18-80) hour doubling time^100^ |
|  | Differentiation | Undifferentiated – organ tissue structure^38^ |
|  | Cell size, diameter floating | 1 pL, 15 µm^86^ |
|  | Start number | 1 10^4^ – 1 10^6 101, 102^ |
| TME | ECM components | Collagen, fibronectin, proteoglycan …^42, 43^ |
|  | ECM % | 20-90%^45, 46^ |
|  | Stroma cell types | Murine endothelial, fibroblasts^40, 49^ |
|  | Stroma cell % | 10-80%^40, 49^ |
|  | Pressure | 0.4-8 kPa (3-60 mmHg)^53^ |
|  | Tumor cord radius | 50-100 µm^57, 103^ |
|  | Capillary diameter | 10 µm^57^ |
|  | Oxygen tension | <1-100 mm Hg (0.1-13%)^60, 104^ |
|  | Glucose concentration | <0.5 - 6 mM^61, 62^ |
| Organism (mouse) - homeostasis | Temperature | 37 ˚C ^64^ |
|  | Blood glucose | 7 mM^65^ |
|  | Blood oxygen tension | 100 mm Hg^66, 67^ |
|  | Hemoglobin concentration, saturation | 2.3 mM, 90-95%^66, 67^ |
|  | Derived oxygen concentration arterial | 8-9 mM |
| Organism (mouse)- pharmacokinetics | Concentration time profile | Drug, dosage dependent. Curve described by peak (C_max_), decline speed (t_1/2_), Area under the Curve (AUC)^69^ |
|  | Drug rounds | 1 to many, daily to weekly^72, 73^ |

**Supplementary table 2.** Examples of cell line-drug combinations of frequent cancers. Xenograft growth inhibition data for these combinations can serve as a starting point for empirical validation of xenografts-on-chip.

| **Frequent cancer** | **Common cell lines^100^** | **Examples of often used drugs^105-107^** |
| --- | --- | --- |
| Colorectal | HCT-116, HT-29, SW620 | 5-Fluoruracil, oxaliplatin, irinotecan*** |
| Lung (non-small cell) | A549, H460 | Cisplatin, docetaxel, erlotinib* |
| Breast** | MCF-7, MDA-MB-231, T-47D | Doxorubicin, paclitaxel, trastuzumab* |

*For targeted drugs erlotinib and trastuzumab target mutation status of the cell line should be checked. **Dependent on hormone receptor status of cell lines, hormone therapy, e.g. tamoxifen, could be an alternative to the drug listed in the table.

*** Irinotecan activity occurs via active metabolite SN38

For every cell-line drug combination a Pubmed (<https://pubmed.ncbi.nlm.nih.gov/>) search can be conducted with keyword combinations such as “xenograft AND oxaliplatin AND (HCT116 OR HCT-116)”. Control and treated xenograft growth (inhibition) can be obtained from the growth graphs in articles. Also the drug dose administered should be obtained. Unless there is a reason for using a specific dose, the median administered drug dose can be used for finding accompanying pharmacokinetic data in mice.

**References:**

[99]Workman P, Aboagye EO, Balkwill F, Balmain A, Bruder G, Chaplin DJ, et al. Guidelines for the welfare and use of animals in cancer research. Br J Cancer 2010;102(11):1555–1577.

[100]Cell Line Metadata [Available from: <https://discover.nci.nih.gov/cellminer/celllineMetadata.do>.

[101]van der Heijden M, Miedema DM, Waclaw B, Veenstra VL, Lecca MC, Nijman LE, et al. Spatiotemporal regulation of clonogenicity in colorectal cancer xenografts. Proc Natl Acad Sci USA 2019;116(13):6140–6145.

[102]Nukatsuka M, Nakagawa F, Takechi T. Efficacy of combination chemotherapy using a novel oral chemotherapeutic agent, tas-102, with oxaliplatin on human colorectal and gastric cancer xenografts. Anticancer Res 2015;35(9):4605–4615.

[103]Primeau AJ, Rendon A, Hedley D, Lilge L, Tannock IF. The distribution of the anticancer drug Doxorubicin in relation to blood vessels in solid tumors. Clinical Cancer Research : an official journal of the American Association for Cancer ResearchClin Cancer Res 2005;11(24 Pt 1):8782–8788.

[104]Carreau A, El Hafny-Rahbi B, Matejuk A, Grillon C, Kieda C Why is the partial oxygen pressure of human tissues a crucial parameter? Small molecules and hypoxia. J Cell Mol Med 2011;15(6):1239–1253.

[105]Van Cutsem E, Cervantes A, Adam R, Sobrero A, Van Krieken JH, Aderka D, et al. ESMO consensus guidelines for the management of patients with metastatic colorectal cancer. Ann Oncol 2016;27(8):1386–1422.

[106]Planchard D, Popat S, Kerr K, Novello S, Smit EF, Faivre-Finn C, et al. Metastatic non-small cell lung cancer: ESMO Clinical Practice Guidelines for diagnosis, treatment and follow-up. Ann Oncol 2018;29(Suppl 4):iv192-iv237.

[107]Cardoso F, Senkus E, Costa A, Papadopoulos E, Aapro M, André F, et al. 4th ESO-ESMO international consensus guidelines for advanced breast cancer (ABC 4)†. Ann Oncol 2018;29(8):1634–1657.
